# Development of Useful Biomaterial for Bone Tissue Engineering by Incorporating Nano-Copper-Zinc Alloy (nCuZn) in Chitosan/Gelatin/Nano-Hydroxyapatite (Ch/G/nHAp) Scaffold

**DOI:** 10.3390/ma10101177

**Published:** 2017-10-17

**Authors:** Juan Carlos Forero, Eduardo Roa, Juan G. Reyes, Cristian Acevedo, Nelson Osses

**Affiliations:** 1 Programa de Doctorado en Biotecnología, Pontificia Universidad Católica de Valparaíso/Universidad Técnica Federico Santa María, Valparaíso 2340000, Chile; juan.forero.o@mail.pucv.cl; 2 Instituto de Química, Facultad de Ciencias, Pontificia Universidad Católica de Valparaíso, Valparaíso 2340000, Chile; eroatoledo@gmail.com (E.R.); juan.reyes@pucv.cl (J.G.R.); 3 Centro de Biotecnología and Departamento de Física, Universidad Técnica Federico Santa María, Valparaíso 2340000, Chile; cristian.acevedo@usm.cl

**Keywords:** scaffold, metallic nanoparticles, chitosan, gelatin, nanohydroxyapatite, bone tissue engineering

## Abstract

Ceramic and metallic nanoparticles can improve the mechanical and biological properties of polymeric scaffolds for bone tissue engineering (BTE). In this work, nanohydroxyapatite (nHAp) and nano-copper-zinc alloy (nCuZn) were added to a chitosan/gelatin (Ch/G) scaffold in order to investigate the effects on morphological, physical, and biocompatibility properties. Scaffolds were fabricated by a freeze-drying technique using different pre-freezing temperatures. Microstructure and morphology were studied by scanning electron microscopy (SEM), glass transition (*T_g_*) was studied using differential scanning calorimetry (DSC), cell growth was estimated by MTT assay, and biocompatibility was examined in vitro and in vivo by histochemistry analyses. Scaffolds and nanocomposite scaffolds presented interconnected pores, high porosity, and pore size appropriate for BTE. *T_g_* of Ch/G scaffolds was diminished by nanoparticle inclusion. Mouse embryonic fibroblasts (MEFs) cells loaded in the Ch/G/nHAp/nCuZn nanocomposite scaffold showed suitable behavior, based on cell adhesion, cell growth, alkaline phosphatase (ALP) activity as a marker of osteogenic differentiation, and histological in vitro cross sections. In vivo subcutaneous implant showed granulation tissue formation and new tissue infiltration into the scaffold. The favorable microstructure, coupled with the ability to integrate nanoparticles into the scaffold by freeze-drying technique and the biocompatibility, indicates the potential of this new material for applications in BTE.

## 1. Introduction

Bone substitutes are becoming a suitable option to reconstruct bone defects instead of autogenous or allogenous bone graft procedures [1]. A requirement for bone tissue engineering substitutes is the development of three-dimensional scaffolds that are biocompatible, biodegradable, and osteoinductive. In addition, they must support cell adhesion, proliferation, and viability [1,2,3].

Chitosan, a natural copolymer of glucosamine and *N*-acetylglucosamine [4], has been widely used as scaffold in tissue engineering because it is biocompatible, non-toxic, reabsorbable, and has antimicrobial properties [5]. Similarly, the water-soluble protein gelatin is used as biomaterial for biomedical applications. Gelatin is obtained from the denaturation of collagen and is suitable as a scaffold because is biocompatible, non-toxic, and reabsorbable [6]. In addition, gelatin has low immunogenicity and contains Arg-Gly-Asp (RGD)-like sequences that promote cell adhesion [7,8]. Importantly, composite scaffolds based on chitosan and gelatin have strong potential to be used in tissue engineering and have been evaluated in different experimental conditions for bone regeneration [9,10].

Further enhancement in the properties of biomaterials for bone tissue engineering can be achieved by including nanoscale particles in the polymeric scaffolds [11,12,13,14,15]. It has been shown that the interaction between nanosized particles and organic polymeric materials may result in improved mechanical and biological properties of the scaffold [13,16,17]. As the main mineral component of bone extracellular matrix (ECM), hydroxyapatite (HA) has been used to increase biocompatibility, osteoconductivity, and osteoinductivity in different scaffolds [18,19]. In this regard, osteoblast function is enhanced by using nano-HA (nHAp) compare to traditional micron-sized ceramic material [14,15]. Other special properties of nHAp are due to its small size and large specific surface area [20,21]. On the other hand, metallic nanoparticles of copper (Cu) and/or zinc (Zn) have been incorporated in scaffolds containing nHAp showing increased antibacterial activity and non-toxicity for osteoprogenitor cells [1,22,23]. Beyond the antibacterial properties of these metal ions, Cu and Zn have been shown to be involved in several aspects of osteoblast activity and bone formation, including, mineralization, stimulation of collagen production, osteoblast cell adhesion, and proliferation [24,25,26,27].

Tripathi et al. previously demonstrated that bio-composite scaffolds containing chitosan/nano-hydroxyapatite/nano-copper-zinc has suitable morphological and physical characteristics for Bone Tissue Engineering (BTE) [28]. To advance in the potential clinical application of scaffolds based in chitosan/nanoparticles further studies are required. In this regard, the focus of our study was to generate a nanocomposite scaffold analyzing manufacturing variables, thermo physical properties and in vivo biocompability. First, we established pre-freezing temperature, concentration, and combination of nanoparticles to prepare the nanocomposite scaffold by a systematic approach. Then, we analyzed cell attachment, cell growth, ALP activity, and cellular infiltration into the scaffolds. Finally, we analyzed the biocompatibility of the best scaffold in vivo. Our results indicate that a Ch/Gel/nHAp/nCuZn (0.5/0.25/0.0625/0.00025% in blend) fabricated to −20 °C of pre-freezing temperature has excellent features to be used in BTE.

## 2. Materials and Methods

### 2.1. Materials

Chitosan powder (Ch) 88% deacetylated (120,000 kDa) was purchased from Quitoquímica (Concepción, Chile), type B bovine Gelatin (G) powder was acquired from Merck KGaA (Darmstadt, Germany), hydroxyapatite-nano powder (nHAp; <200 nm), Cu-Zn alloy nanoparticles (nCuZn; <150 nm) and MTT (3,4,5-dimethylthiazol-2yl-2,5-diphenyl-2H-tetrazoliumbromide) were purchased from Sigma-Aldrich (St. Louis, MO, USA), Mouse Embryonic Fibroblasts (MEFs) were obtained from ATCC (Manassas, VA, USA), and Dulbecco’s modified Eagle’s medium (DMEM) was acquired from Hyclone (Chicago, IL, USA). Fetal bovine serum (FBS) was purchased from GIBCO (Waltham, MA, USA). All other reagents and solvents were obtained from commercial suppliers. All aqueous solutions were prepared with ultrapure water (>18.2 MΩ-cm) from PURELAB classic ELGA Milli-Q system (Paris, France).

### 2.2. Preparation of Ch/G Scaffolds

The scaffolds were prepared using the freeze-drying method: Ch (1% *w*/*v*) and G (1% *w*/*v*) solutions were dissolved in acetic acid solution (100 mM) separately with stirring at 50 °C. The Ch/G blend was mixed at a 2:1 ratio respectively. Subsequently, the blend was cross-linked with different glutaraldehyde (GTA) aqueous solutions at 0.02%, 0.06%, and 0.10% final concentrations. The solutions were stirred for 1 h and poured into standard cell culture dishes (60 × 15 mm). The dishes were stored under ambient conditions overnight to achieve complete crosslinking. Subsequently the dishes were incubated at 37 °C for 24 h. Thereafter, the scaffold solutions were pre-frozen sequentially at −20 °C, −80 °C, or −196 °C for an additional 24 h, and the scaffolds were lyophilized. The lyophilized scaffolds were again cross-linked in their respective glutaraldehyde solution for 1 h. Subsequently, the scaffolds were immersed in 4 mL of 0.1 M glycine solution for 24 h to block the excess aldehyde residues [29]. The scaffolds were rinsed three times with ultrapure water. The freeze-drying procedure (freezing and lyophilization) was repeated to obtain a finished material.

### 2.3. Preparation of Nanoparticle Solutions

The nanoparticle solutions were used to make the nanocomposite scaffolds. For that, Ch, G and nanoparticle solutions were then mixed in proportion of 2:1:1, respectively. Two kind of nanoparticles were used (nHAP and nCuZn). The nHAp solutions were prepared in concentrations of 0%, 0.25%, and 0.50%, and nCuZn solutions in concentrations of 0% and 0.001%. In addition, solution combinations of both nanoparticles were prepared. The scaffold without nanoparticles was obtained when the nanoparticle solution is prepared at 0%.

### 2.4. Scaffold Characterization

#### 2.4.1. Microstructure, Porosity and Roughness

The structural morphology, nanoparticle distribution, and surface elemental analysis of the scaffold were performed by light microscopy, scanning electron microscopy (SEM; Carl Zeiss, EVO MA 10, Oberkochen, Germany), and energy dispersive spectroscopy (EDS) at 25 kV acceleration voltages and a Sirius SD (Silicon Drift) EDS detector (Oxford, X-Act, Abingdon, UK). The pore size was estimated using SEM images, counting a minimum of 100 pores from different places on the cross section of the scaffolds. The pore sizes were analyzed by using ImageJ Software (NIH, Bethesda, MD, USA). For the measure of pore size in ImageJ, we set the scale in the image with bars of known distance. After that, with freehand selection, we trace the outline of the pore and we measure the area in micrometers. The porosity of scaffolds was measured in triplicate using a gas pycnometer (Multivolume 1305, Micrometrics, Norcross, GA, USA) with helium as the displacement gas. Scaffolds surface roughness was estimated by processing a series of stereoscopic microscopy images acquired in different regions of the scaffold surface (Mag. 100×). Arithmetic roughness (Ra), mean roughness (Rq) and 3D surface plot were determined from different images obtained from three independent experiments using the “Roughness Calculation Plugin” and “3D surface plot plugin”, respectively, from ImageJ Software, which determines the surface peaks and valleys altitude to calculate roughness values.

#### 2.4.2. Determination of Glass Transition Temperature (*Tg*)

The glass transition was analyzed by using a differential scanning calorimeter (DSC1 STARe System, METTLER-TOLEDO, Schwerzenbach, Switzerland). A sample of 10 mg was hermetically sealed in a stainless steel pan of 100 μL. The thermal scanning conditions were as follows: heating from −20 to 130 °C at 10 °C/min, holding at 130 °C for 1 min, cooling from 130 to −20 °C at 20 °C/min, holding at −20 °C for 2 min, and reheating to 130 °C at 10 °C/min. The DSC was previously calibrated using indium as a standard, and an empty pan was used as reference. The glass transition temperature (*T_g_*) was determined as the midpoint of the change in heat capacity (Cp) observed in the first heating scan. The value of ∆Cp (variation in heat capacity between the rubbery and glassy states) was expressed based on dry mass of the sample.

### 2.5. Cell Culture Studies

#### 2.5.1. Cell Seeding and Culture on Scaffolds

Mouse embryonic fibroblasts (MEFs) were cultured in 60 × 15 mm dishes (Corning, Corning, NY, USA) in DMEM with 10% Fetal Bovine Serum containing l-glutamine and penicillin-streptomycin at 37 °C in air containing 5% CO_2_. The scaffolds, with a size of 8 mm of diameter and 2.5 mm of thickness, were disinfected by immersion in ethanol 70% (*v*/*v*) for 24 h. Then, the polymer was washed and hydrated for 2 h with PBS prior to cell seeding; thereafter, scaffolds were placed in a 24-well cell culture plate. Then, 2 × 10^4^ cells/scaffold were seeded in a volume that soaked the scaffold and were incubated for 3 h. Five hundred μL of culture medium was added into each well. After 24 h, the scaffolds were changed to new culture wells in order to analyze only the cells growing into the scaffolds. Empty scaffolds (without cells added) were treated in the same manner and used as controls.

#### 2.5.2. Cell Attachment and Proliferation

Cell adhesion and proliferation rates were estimated by MTT assay. For cell adhesion, the cells were loaded onto the scaffold and left for 24 h. After that, the scaffolds were removed and the attached biomass was measured at 570 nm and estimated by the following equation:

$$Cell\;adhesion:\frac{OD570s}{OD570t}\times 100\%$$

where OD570*s* and OD570*t* are the measured optical densities for cells present in the scaffold and the total cells cultured in the plate and scaffold respectively. To determinate the proliferation rate, the biomass into the nanocomposite was measured after 4, 24, 48, and 72 h of cell culture. The percentage of cell growth into the scaffolds was calculated from the following equation:

$$Cell\;growth:\frac{OD570f}{OD570i}\times 100\%$$

where OD570*f* represents the final optical densities of cell culture and OD570*i* is the initial optical densities from first 4 h of cell culture. In addition, H&E staining was used for microscopic examination of distribution and morphology of cells allowed to growth into the scaffolds for 7 and 14 days.

#### 2.5.3. Alkaline Phosphatase Activity

MEF cells were culture in osteogenic medium containing 50 μg/mL ascorbic acid, 10 mM β-glycerolphosphate and 0.1 μM dexamethasone. For quantification of cell alkaline phosphatase (ALP) activity, cell lysate was incubated with One-Step NBT-BCIP solution (Thermo Fisher Scientific, Waltham, MA, USA) at 37 °C for 2 h, and the absorbance was read at 570 nm. A BCA assay was performed to determine protein concentration. 

### 2.6. In Vivo Biocompatibility Assay

#### 2.6.1. Surgical Procedure

To evaluate in vivo tissue interactions of nanocomposite scaffold selected from in vitro studies, a subcutaneous biosecurity assay was performed. Two male rabbits (Oryctolagus cuniculus), each weighing approximately 2.5 kg, were used for animal experiments. Rabbits were kept in an individual cage and were housed in a temperature-controlled facility. All the procedures were performed in accordance with the rules laid down by the Consortium for Developing a Guide for the Care and Use of Agricultural Animals in Agricultural Research and Teaching, performed by veterinarians and approved by the Institutional Bioethics Committee of Pontificia Universidad Católica de Valparaíso, Chile (Cod: BIOEPUCV-A100-2015). None of the authors served in this committee. Rabbits were anesthetized with ketamine/xylasine (10 mg/kg and 2 mg/kg respectively). Each animal received two subcutaneous implants with 30 mm in diameter; two longitudinal incisions of about 25 mm were made through the full thickness of the skin of right and left dorsum. Into the right incision, we placed the nanocomposite scaffold, and the control without nanoparticles was placed into the left. Upon implantation of the scaffolds into the dorsum, the cut was sutured using a reabsorbable suture material. Nanocomposites implanted were examined four week after surgery. A minimum of three areas per implant were examined.

#### 2.6.2. Histochemistry

To obtain the biopsy of subcutaneous implants, animals were sacrificed after four weeks, and the implants were fixed in 4% paraformaldehyde in 0.1 M phosphate buffer, PH 7.4, at 4 °C for 24 h until further processing for histological analysis. The conventional H&E staining was used for microscopic examination of paraffin embedded histological sections (5 μm thickness). 

### 2.7. Statistical Analysis and Experimental Design

Experiments were performed in triplicate unless otherwise indicated. The data are expressed as the mean ± standard deviation. Basic statistical analyses (*t*-test, ANOVA and two-way ANOVA) were performed by using statistical tools of the software GraphPad Prism version 4.0 (San Diego, CA, USA). Differences at the level of *p* < 0.05 were accepted as significant. The experimental design was performed using Design Expert 7.0 software (Statease, Minneapolis, MN, USA). A two-factor design based on Central Composite Face Centered (CCF) was run per duplicate with three central points. Relative effects of the factors on response (*T_g_*) were identified from 3D surface plots.

## 3. Results

### 3.1. Ch/G Scaffold and Nanocomposite Scaffold Characterization

**Figure 1:**
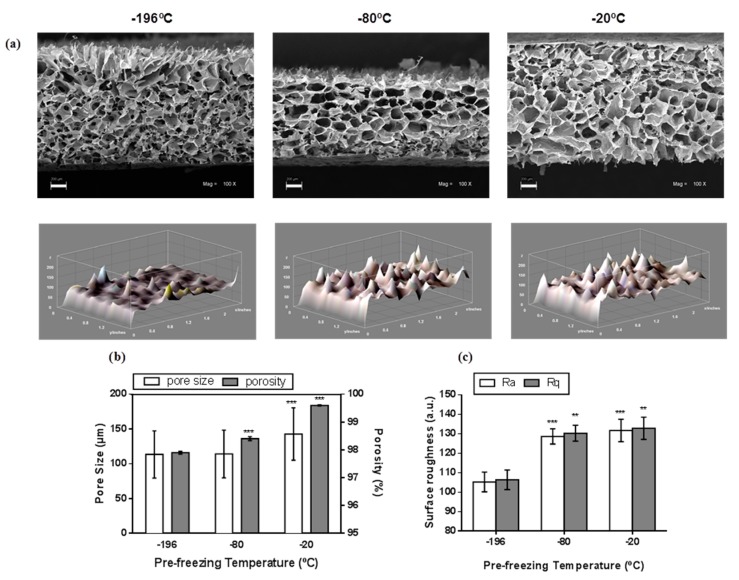
Morphological characterization of Ch/G scaffolds was analyzed by scanning electron microscopy (SEM) for pore size, gas picnometry for porosities, and photomicrography analyses for surface roughness: (**a**) Cross-sectional images of scaffolds generated at different pre-freezing temperatures (upper row). Microstructure and pore distribution are appreciated (Mag. 100×, scale bar: 200 micrometers). Lower row show 3D surface plot of scaffolds; (**b**) Pore size and porosity measurements; (**c**) Surface roughness is represented by arithmetical mean deviation (Ra) and root mean square deviation (Rq). Values are presented as mean ± S.D and corresponds to collected data from three independent experiments (pore size *n* = 100; porosity and surface roughness *n* = 3). ** *p* < 0.01, *** *p* < 0.001 compared to −196 °C. One-way ANOVA was used for comparison.

**Figure 2:**
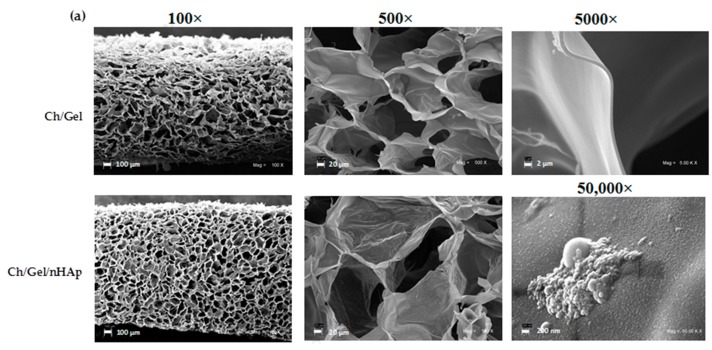

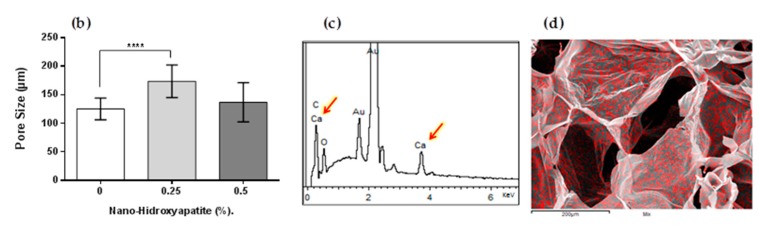
SEM photomicrographs, pore size, and energy dispersive spectroscopy (EDS) analysis of nano-composite scaffold (Ch/G/nHAp). (**a**) Comparison of microstructure of scaffold and nanocomposite scaffold in different magnifications; (**b**) Pore size measured at different nanohydroxyapatite (nHAp) concentrations; (**c**) Calcium peaks detected in nanocomposite scaffold (arrows); (**d**) Red points show the calcium distribution on nano-composite surface. In Figure 2b, data are presented as mean ± SD obtained from three independent experiments. **** *p* < 0.0001 compared to control group. One-way ANOVA was used for comparison.

The SEM analysis of the scaffolds prepared without nanoparticles at different pre-freezing temperatures revealed a 3D microstructure with pores of different sizes and shapes (Figure 1a, upper row) including changes in the surface (Figure 1a, lower row). Gas pycnometry and SEM analyses showed that increasing the pre-freezing temperature during scaffold preparation produced an increase of porosity from 97.8 to 99.5% and the pore size from 113 to 143 μm respectively (Figure 1b). In addition, an increase of about 30% in roughness parameters (arithmetic and mean roughness) were obtained by increasing the pre-freezing temperature from −196 to −20 °C (Figure 1c). Therefore, the pore size, porosity and surface roughness can be modulated by regulating the pre-freezing temperature during Ch/G scaffolds synthesis. On the other hand, nHAp addition into polymeric Ch/G scaffold fabricated at −20 °C of pre-freezing temperature has no effect in the overall morphology (Figure 2a). However, an increase of 173% in the pore size was observed at a 0.25% concentration of nanoparticles (Figure 2b). Energy dispersive spectroscopy (EDS) spectra showed energy peaks of high intensity corresponding to the presence of calcium (Figure 2c). Although some nanoparticle clusters were detected on the surface of the pore wall (Figure 2a, lower row), analysis of calcium by EDS showed a homogeneous distribution throughout the scaffold in intimate association with the Ch/G matrix (Figure 2d). Thus, a uniform Ch/G/nHAp scaffold was achieved using −20 °C of pre-freezing temperature without affecting morphological properties.

### 3.2. Influence of Nanoparticle Content and Crosslinker on Glass Transition Temperature (T_g_)

**Figure 3:**
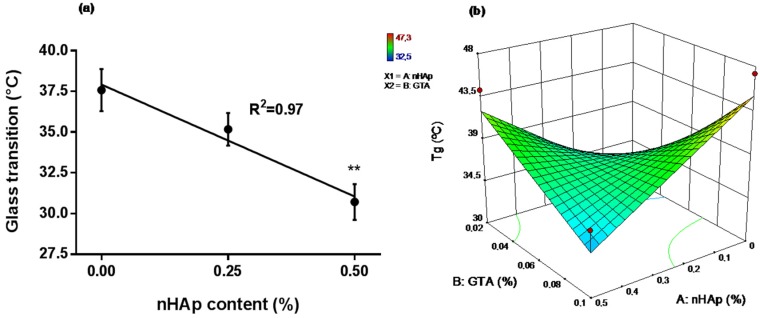
Influence of nHAp and glutaraldehyde (GTA) concentration on *T_g_* and application of central composite face-centered design. (**a**) *T_g_* evaluated by DSC in function of nHAp content on Ch/G scaffolds; (**b**) 3D surface plot of the influence of nHAp and GTA contents on *T_g_*. Data obtained from three independent experiments.

To evaluate the thermophysics characteristics at or below 37 °C, the glass transition of nanocomposite scaffolds was analyzed by DSC. Figure 3a shows that *T_g_* decreases from 37.57 °C (without nHAp) to 30.71 °C by incorporation of nHAp (0.5% *w*/*v*). Thus, the glass transition properties of the polymeric scaffold were reduced by nanoparticle addition. To predict the simultaneous influence of cross-linker (GTA) and nHAp content on *T_g_* after nanocomposite scaffold synthesis, a CCF analysis was carried out. The analysis showed that nHAp and GTA content independently were not a significant influence on *T_g_*, but the nanoparticle content showed significant effects on endpoint and ∆*Cp* (Table 1). ∆*Cp* significant results shown that nHAp and GTA content independently modulates the amount of heat required to increase the temperature of polymeric matrix. ∆*Cp* significant results shown that nHAp and GTA content independently modulates the amount of heat required to increase the temperature of the polymeric matrix. The influence of nHAp and GTA on *T_g_* (Figure 3b) showed that higher nHAp (0.5%) and GTA content (0.1%) decrease *T_g_*, while no addition of nanoparticles increases the *T_g_* maintaining the same GTA concentration (0.1%). Based on these data and the laboratory handling process, the final concentrations of the components were selected to prepare the nanocomposite scaffolds for in vitro cellular studies (Table 2).

### 3.3. Cell Attachment, Proliferation and Alkaline Phosphatase Activity

**Figure 4:**
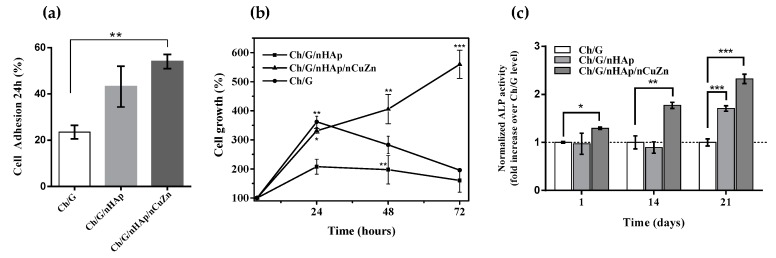
In vitro analysis of cells cultured on nano-composite scaffolds. (**a**) Percentage of osteoblastic lineage (MEF) cells adhesion at 24 h post-seeding and (**b**) Kinetics of cell growth; (**c**) alkaline phosphatase (ALP) activity of MEF cells plated in different scaffolds quantified and normalized to Ch/G scaffold levels. In Figure 4a,b, data are presented as percentage ± SD; (**a**) ** *p* < 0.01 compared to Ch/G scaffold; (**b**) * *p <* 0.05, ** *p* < 0.01, *** *p* < 0.001 compared to initial time (4 h). In Figure 4c, data are presented as fold increase, and * *p* < 0.05, ** *p* < 0.01, *** *p* < 0.001 compared to Ch/G scaffold group. One-way ANOVA was used for comparison. S. Data corresponds to triplicate determinations from three independent experiments.

Quantification of cell adhesion reveals a 2-fold increase in attached cells on nanocomposite scaffolds (nHAp and nHAp/nCuZn) compare to Ch/G scaffold (Figure 4a). On average, the highest values were obtained for the scaffold containing Ch/G/nHAp/nCuZn where 54 ± 3 percent of plated cells are attached after 24 h. In addition, a significant rise in cell growth was observed in cells cultured on a Ch/G/nHAp/nCuZn scaffold, increasing the cell growth 5-fold after 72 h of culture (Figure 4b). On the other hand, it has been shown that a 3D environment promotes differentiation of MEFs into osteoblasts-like cells [30], as well as Ch/nHAp matrices upregulates ALP, a sign of osteoblastic differentiation [13]. Therefore, we analyzed the ALP activity of cells plated on control (Ch/G) and nanocomposite scaffolds. Figure 4c shows that after 21 days, ALP activity was enhanced in the Ch/G/nHAp and Ch/G/nHAp/nCuZn nanocomposite scaffolds by 1.7- and 2.3-fold with respect to the Ch/G scaffold. Taken together, our data indicates that the incorporation of nCuZn alloy nanoparticles in the Ch/G/nHAp scaffolds provides a favorable environment for cell adhesion, growth, and differentiation.

### 3.4. Biocompatibility Analysis of Ch/G/nHAp/nCuZn Scaffold

**Figure 5:**
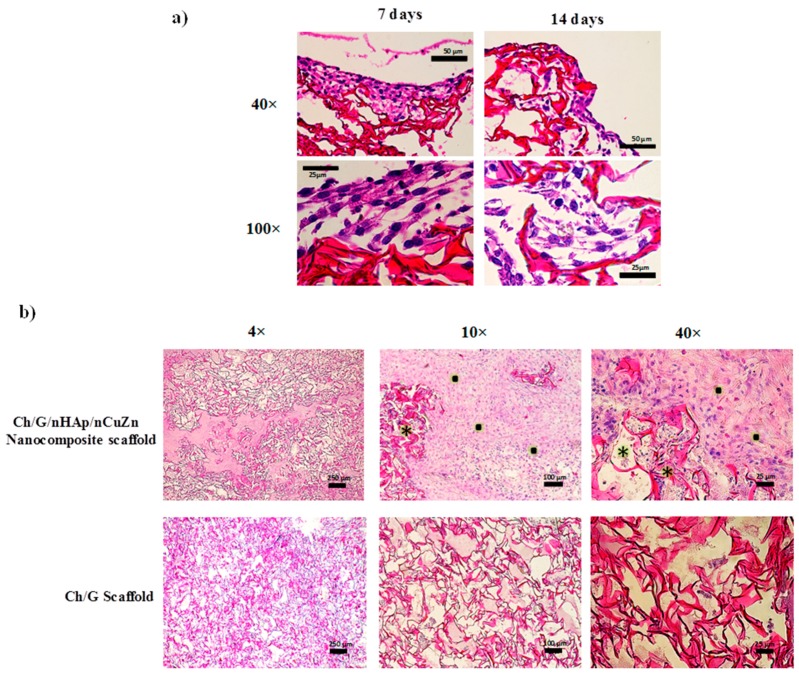
(**a**) Cross-sectional histological photomicrographs from the Ch/G/nHAp/nCuZn nanocomposite scaffold stained with hematoxylin/eosin at 7 days and 14 days after MEF cell culture; (**b**) Hematoxylin/eosin stained sections of scaffolds from in vivo biocompatibility assessment after four weeks post-implant; tissue ingrowth in Ch/G/nHAp/nCuZn nanocomposite scaffold (4× and 10×). Formation of granulation tissue (●) and degradation of scaffold matrix (*) are indicated. Ch/G implant was used as a control (lower row).

In order to evaluate the biocompatibility of the Ch/G/nHAp/nCuZn scaffold, we first performed histochemical analysis of sectioned paraffin blocks (5 μm thick) of the scaffold containing MEF cells. Figure 5a shows the cross-section of nanocomposite scaffold seeded with MEF cells after 7 and 14 days of culture. Comparing the stained histology images, during the first week, cells were present mainly at the nanocomposite surface (Figure 5a, left column), whereas at 14 days, an appreciable infiltration of cells into the pores was observed (Figure 5a, right column). Closer examination showed elongated cells in close contact with the scaffold (Figure 5a, lower row). These results are in agreement with the capacity of the Ch/G/nHAp/nCuZn scaffold to support cellular adhesion and proliferation (Figure 4). 

The aim of in vivo biocompatibility assessment was to investigate whether the rabbit subcutaneous tissue accepted the Ch/G/nHAp/nCuZn nanocomposite scaffold and to study tissue ingrowth. The biopsy specimens were taken after four weeks of implant. The nanocomposite scaffold showed new tissue infiltration and large number of cells in the matrix (Figure 5b, upper row). Granulation tissue was observed mainly in the edge areas (Figure 5b, upper row 10× and 40×). On the other hand, scaffold without nanoparticles showed poor tissue infiltration (Figure 5b, lower row). These results showed that the nanoparticle content (nHAp/nCuZn) helps cell migration and the formation of granulation and connective tissue.

## 4. Discussion

The application of nanotechnology in tissue engineering nowadays has led the use of different nanoparticles for development of novel scaffolds. In this regard, it is important to evaluate the influence of nanoparticles on morphological, physical, and biological properties before the potential use of viable implants that involves the mimicking of functional tissue. In this report, we evaluated morphological and physical effects of nHAp addition in a polymeric blend and reported the biological effects of nHAp and nCuZn alloy into Ch/G scaffold. In the synthesis of polymeric scaffolds and nanocomposite scaffolds by a freeze drying technique, pore size at three different pre-freezing temperatures was greater than 100 μm (Figure 1), a value accepted as the minimum pore size required for bone tissue regeneration [31]. Generally, a low rate of nucleation and a low rate of crystal growth are caused by freezing at relatively high temperatures. Therefore, this reduces the number of large crystals. As a result, the average pore diameter obtained by freeze-drying increases with raising pre-freezing temperature [32]. Thus, pre-freezing temperature is an important factor to affect the mean pore diameters of the final scaffolds. Besides, the scaffolds had an interconnected porous structure with pore sizes that are known to be able to support bone and vascular ingrowth [33]. On the other hand, high porosities are important to allow cell infiltration into the scaffolds, hence our porosity values (Figure 1) are ideal for the scaffold to interact and integrate with a host tissue [34]. 

Microtopography in bone tissue engineering scaffolds plays an important role in bone-implant contact (BIC). An increase of surface roughness of implants enhances the process of osseointegration, which increases the bone formation and increases BIC [35]. The results of the scaffolds’ surface roughness estimated by imagenological analysis shows an increase when the pre-freezing temperature is elevated (Figure 1). Therefore, these values might be associated with the porosity. This effect might be a consequence of the increased porosity observed during the increase of pre-freezing temperature. A positive association between porosity and surface roughness has been observed in different materials designed for tissue engineering, including titanium implants [36], polystyrene fibers [37], and Ultra High Molecular Weight Poly-Ethylene [38].

As seen in Figure 2, nanoparticle addition to a polymeric scaffold showed minor changes in pore size examined by SEM. However, these nanoparticles modified the pore wall surface, providing roughness to the microstructure. This property is significant because is well-known that attachment, proliferation and differentiation of anchorage dependent bone forming cells are improved by the roughness of pore surface [36]. Thus, additional microstructural roughness can be obtained by including nHAp independently of changes in porosity. 

It has been reported that inorganic nHAp in polymeric matrices containing Ch and/or G exhibit strong chemical interactions via covalent bonding, ion-dipole interactions, and complexation of Ca^2+^ ions with polymer amino, acetylamino, and hydroxyl groups [33,39]. Our EDS analysis showed that calcium is scattered throughout the scaffold (Figure 2f). The close association of calcium phosphate nanoparticles with chitosan and direct chemical bonding between organic and inorganic phases could limit the ability of the nano-particles to migrate away from the implant and result in improved mechanical properties as well as decreased tissue damage [33]. 

Changes in the *T_g_* as a function of the filler content have been reported [17] for polymer composites containing a wide variety of fillers and polymers. The *T_g_* evaluated in CCF design performed in this study, showed the influence of nanoparticle content and GTA concentration (Figure 3b). The nHAp interaction with Ch/G blend has not been clearly studied; however, changes of the polymer *T_g_* due to nanoparticles content have been fairly well studied [40]. Both increases and decreases on *T_g_* have been reported depending upon the interaction between the matrix and the particle [41]. The decreasing of *T_g_* can be due to an increase in polymer chain mobility by the absence of interaction between nanoparticles and the polymeric solution [42]. Moreover, the presence of nanoparticles in the cross-linked copolymer could yield a crosslink density change over the composite because preferential interactions of glutaraldehyde with the nanoparticle surface or interruption of the crosslink density due to confinement effects [41]. It is generally accepted that the crosslink density is a critical factor influencing the thermal properties of nanocomposites [43,44]. On the other hand, as expected for a cross-linked network, an increase of GTA concentration increases *T_g_* at the same time that the concentration of nanoparticles decreases. It is known that local molecular packing due to crosslinking changes results in a decrease in free volume, leading to an increase in *T_g_* [45]. It is important to note that nHAp incorporation into copolymer (Ch/G) in our fabrication process lead to decreasing of *T_g_* below 37 °C. In this regard, tissue engineering scaffolds with *T_g_* of 37 °C (physiological temperature) or lower is desirable since scaffolds having *T_g_* higher than the physiological temperature tend to be brittle and can fracture when subjected to stress in use [46]. 

Analysis of in vitro cellular adhesion (Figure 4a) is in agreement with previous studies focused on cell-material interactions. Different synthesized scaffolds have shown that nano-sized ceramics and nHAp increases cell adhesion [14]. Qiu et al. (2013) showed that the use of Silica-Hydroxyapatite (Si-Hap) discs improve the adhesion and proliferation of bone mesenchymal stem cells [47]. The incorporation of nCuZn alloy nanoparticles into Ch/G/nHAp scaffold improves cell adhesion and proliferation (Figure 4a,b). The mechanisms for these effects need further studies, but incorporation of nCuZn in Ch/nHAp scaffolds has shown to increase swelling, decrease degradation and increase protein adsorption [28]. Those properties could provide better cell-material interaction enabling cell adhesion and proliferation. Importantly, Ch/G/nHAp/nCuZn scaffolds also increased ALP activity in cells non-committed with the osteoblastic lineage (MEF), suggesting that has osteogenic properties. ALP activity is a widely accepted marker for osteoblastic phenotype, and higher levels reflect a more differentiated stage. In this regard, several studies have shown that the presence of nano-hydroxyapatite particles in scaffolds based on chitosan provide better bone bioactivity [48] and increased ALP activity [49]. In addition, Zn contributes to enhance ALP activity in different cells culture, such as human bone marrow stromal cells (hBMSCs) [50], mesenchymal stem cells [51], and murine preosteoblast cell line (MC3T3-E1) [52]. On the other hand, the role of Cu^2+^ ions in osteogenic differentiation is not well known. However, mesenchymal stem cells seeded on titanium implants containing copper in low concentrations on the surface, shows a higher activity of ALP compare to titanium alone, indicating that copper had a positive effect on osteogenic differentiation [53]. Similarly, Wu et al, showed that hBMSCs cultured in a Copper-containing mesoporous bioactive glass scaffolds (Cu-MBG) display a slight trend of increased in ALP activity compared to those without Cu ions [54]. On the other hand, the low nCuZn alloy concentration used in Ch/G/nHAp scaffold had no cytotoxic effects. An in vitro cytotoxic study of nCuZn alloy was reported by Kumbıçak et al.: their finding showed an average IC50 values were 4.55 μg/mL and 4.66 μg/mL in human lung epithelial cells (BEAS-2B) [55]. These concentrations are very high with respect to the lower concentrations used in our study (100 ppm).

Good biocompatibility is required in the development of bone implant material. Accordingly, we performed histological analysis of cells plated on the Ch/G/nHAp/nCuZn scaffold in vitro, as well as acellular subcutaneous implant of the nanocomposite in rabbits. We observed that the scaffold had no obvious toxic effects on cells cultured for 14 days. Similarly to the behavior of fibroblast cells plated on Ch/G/hyaluronic acid scaffolds [56], in the first days, we observed cells mainly on the surface of the scaffold, and after a week, cells were abundant in the middle zone, suggesting that the Ch/G/nHAp/nCuZn scaffold promotes the infiltration of cells. In agreement, the Ch/G/nHAp/nCuZn scaffold implanted for four weeks showed a high cell growth inside compared to the Ch/G scaffold. Whether the growing tissue is due to the presence of nHap or nCuZn is actually unknown, but subcutaneous implantation of hydroxyapatite-coated cellulose sponges in rats produce early inflammatory cell recruitment and later promotes growth of granulation tissue [57]. The granulation tissue that grew inside the Ch/G/nHAp/nCuZn nanocomposite scaffold is an important condition that provides a vascularized network for subsequent deposition of collagen and tissue regeneration [58].

## 5. Conclusions

In summary, this study shows that the composition and fillers of ceramics blended with metallic nanoparticles play an important role in the morphological, physical, and biocompatibility properties of nanocomposite scaffolds. Highly porous and open interconnected pore structural scaffolds were fabricated through a freeze drying technique. In vitro studies showed that the Ch/G/nHAp/nCuZn scaffold was suitable for MEF cell culture based on adhesion and cell growth assays. These results were confirmed with histological cross-sections that showed good cellular confluence and infiltration into the pore walls. Moreover, nanocomposite scaffold used for in vivo assays promotes the growth of surrounding tissues and induces a vigorous proliferation of granulation tissue and subsequently new connective tissue formation. We have demonstrated that the Ch/G/nHAp/nCuZn nanocomposite scaffolds developed in this study can fulfill many of the requirements of prospective candidates for bone tissue engineering applications.

## Figures and Tables

**Table 1:** Results of the CCF experimental design expressed as *p*-value.

Factor	Glass Transition (First Scan)			
	Onset	*Tg*	Endpoint	∆*Cp*
A	0.62	0.32	0.06	0.05 *
B	0.19	0.12	0.04 *	0.03 *
A × B	0.16	0.06	0.01 *	0.06

(A) GTA, (B) nHAp; * Statistically significant effect (*p* < 0.05).

**Table 2:** Nano-composite designation, composition and final concentrations in blends.

Scaffold Designation	Composition			
	Chitosan (Ch) 1%	Gelatin (Gel) 1%	nHAp 0.25%	nCuZn Alloy 0.01%
	Final Concentrations in Blend (%)			
Ch/Gel	0.5	0.25	-	-
Ch/Gel/nHAp	0.5	0.25	0.0625	-
Ch/Gel/nHAp/nCuZn	0.5	0.25	0.0625	0.00025
